# When We Were Young, Tom

**Published:** 1899-09

**Authors:** 


### When We Were Young, Tom.

Time has flown since we were young, "Tom,"
It seems not long ago
When we were like the other boys,
So full of push and go.

But now we're getting old, "Tom,"
The frost is on our brow;
We do not run and jump as then,
But sit much longer now.

Our vision, too, is growing dim—
We spectacles must wear;
And silken cap upon our head
Where once we wore our hair.

We both have been quite busy men,
We've seen both ups and downs—
We've seen the world and measured it,
With all its smiles and frowns.

You've seen some things I have not seen,
And I, that you have not,
You've borne your load, as I have mine,
Which neither has forgot.

To do our duty, both have tried
As each has seen the light,
Though dark sometimes has seemed the way,
We've tried to do the right.

In outer life, in inner self,
The first, the world to show;
The inner self the crowd knows not—
That God alone can know.

Then still, friend "Tom," will you and I,
While yet 'tis called "to-day,"
Strive for the best till night shall come,
To lay aside the clay?

When outer life shall be cast off,
And we shall fly away
With inner self, for further use
In God's eternal day. —*G. H. C.*

